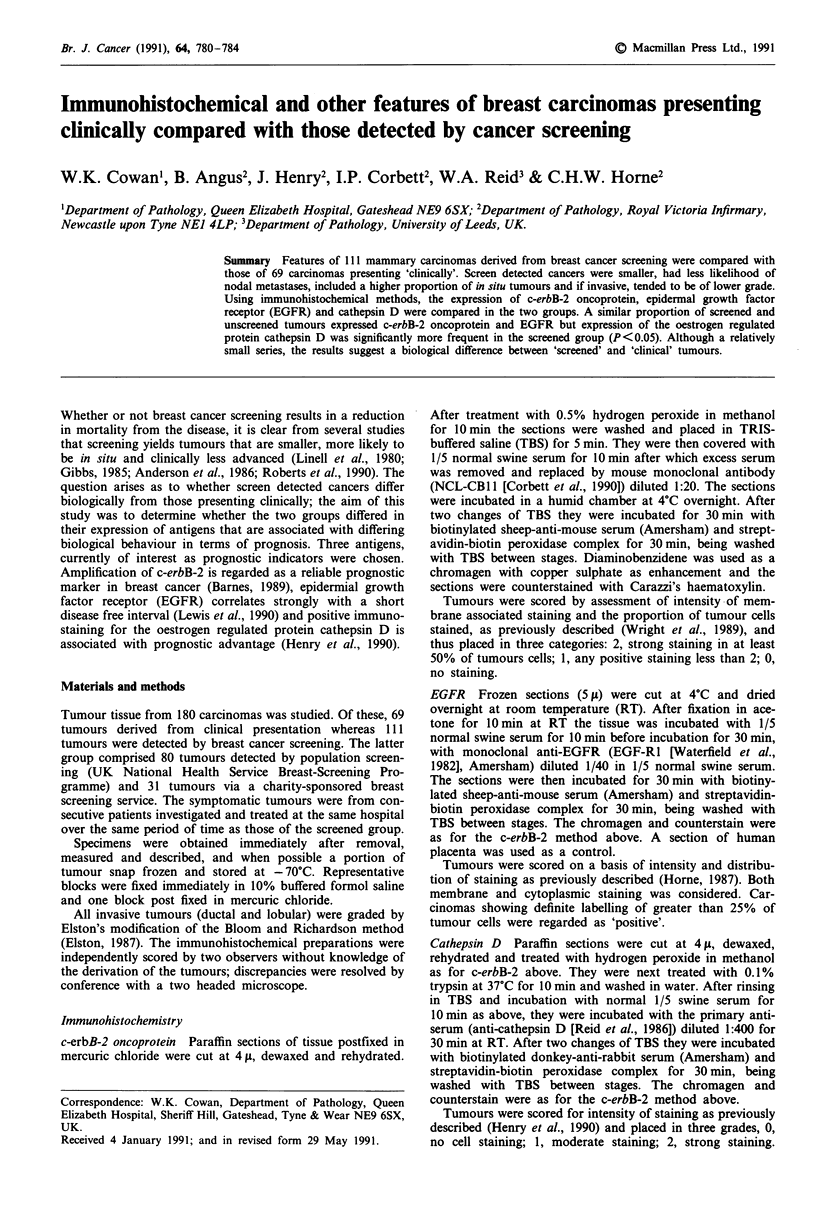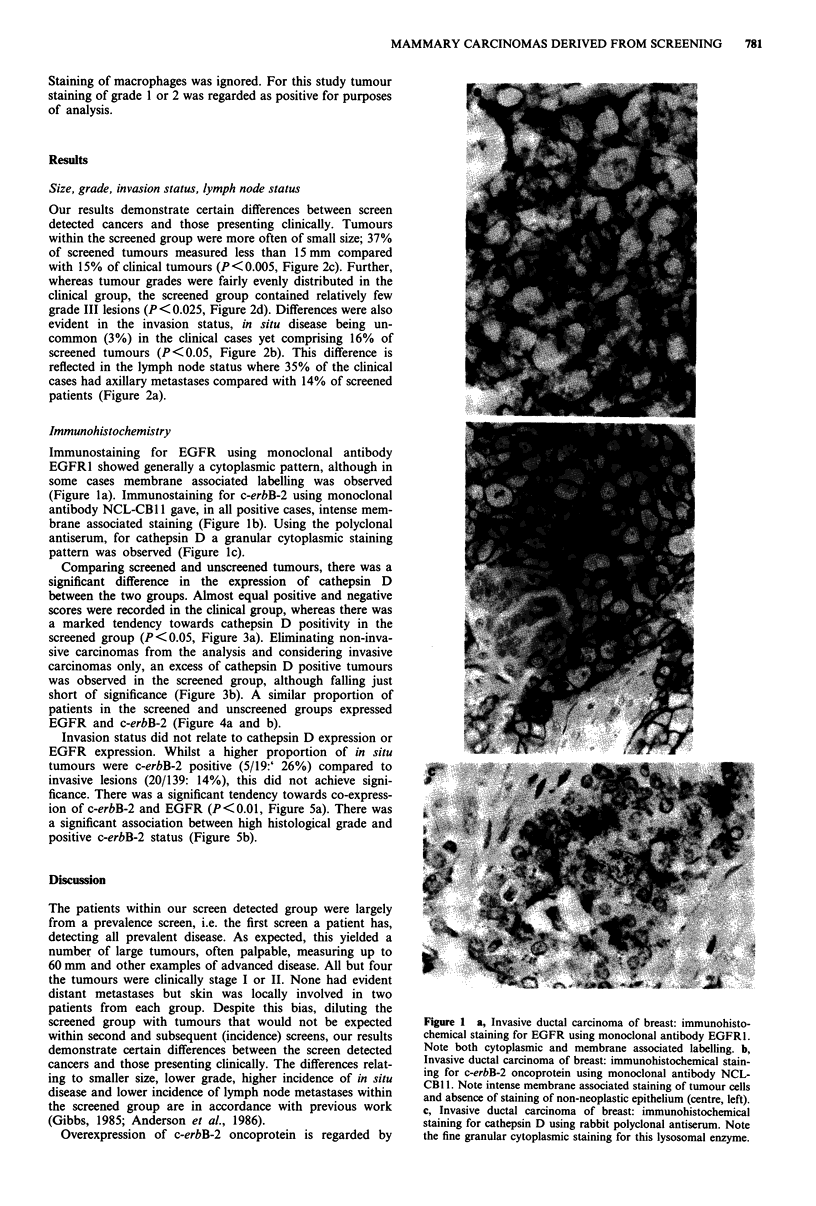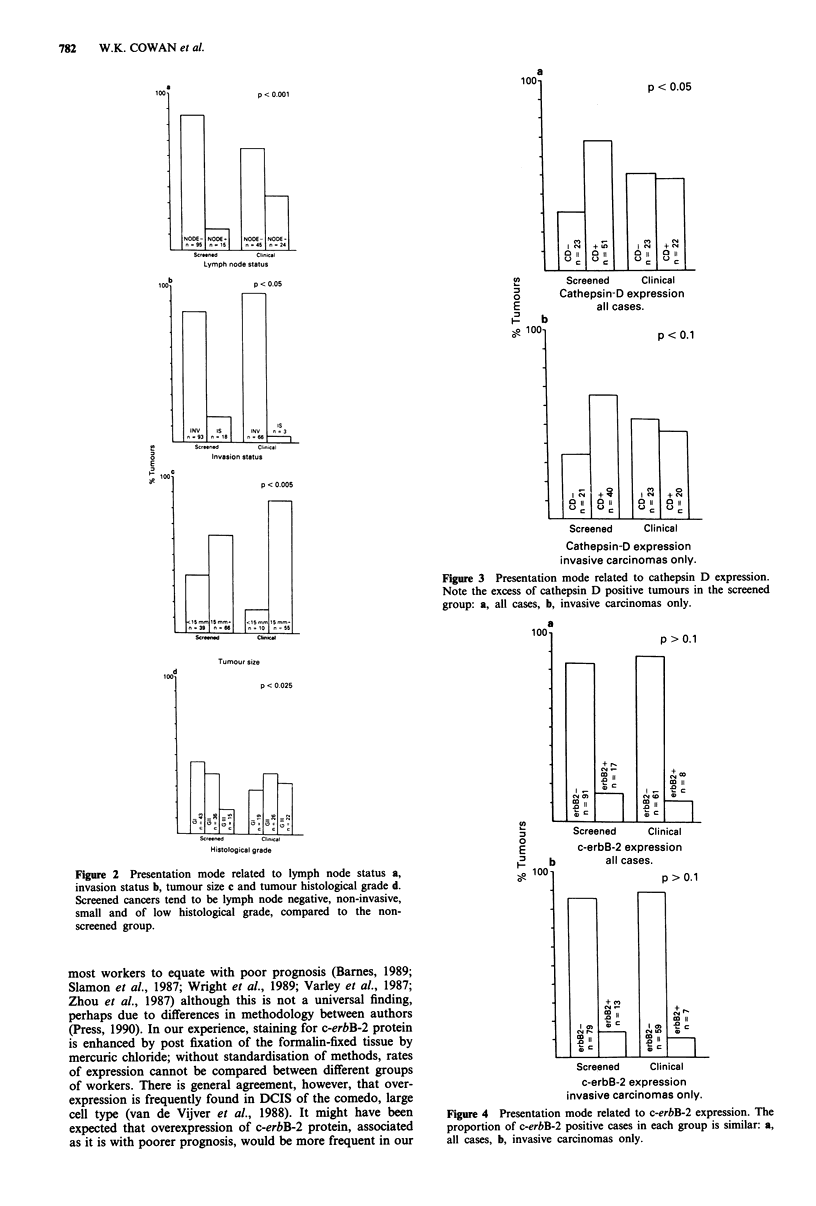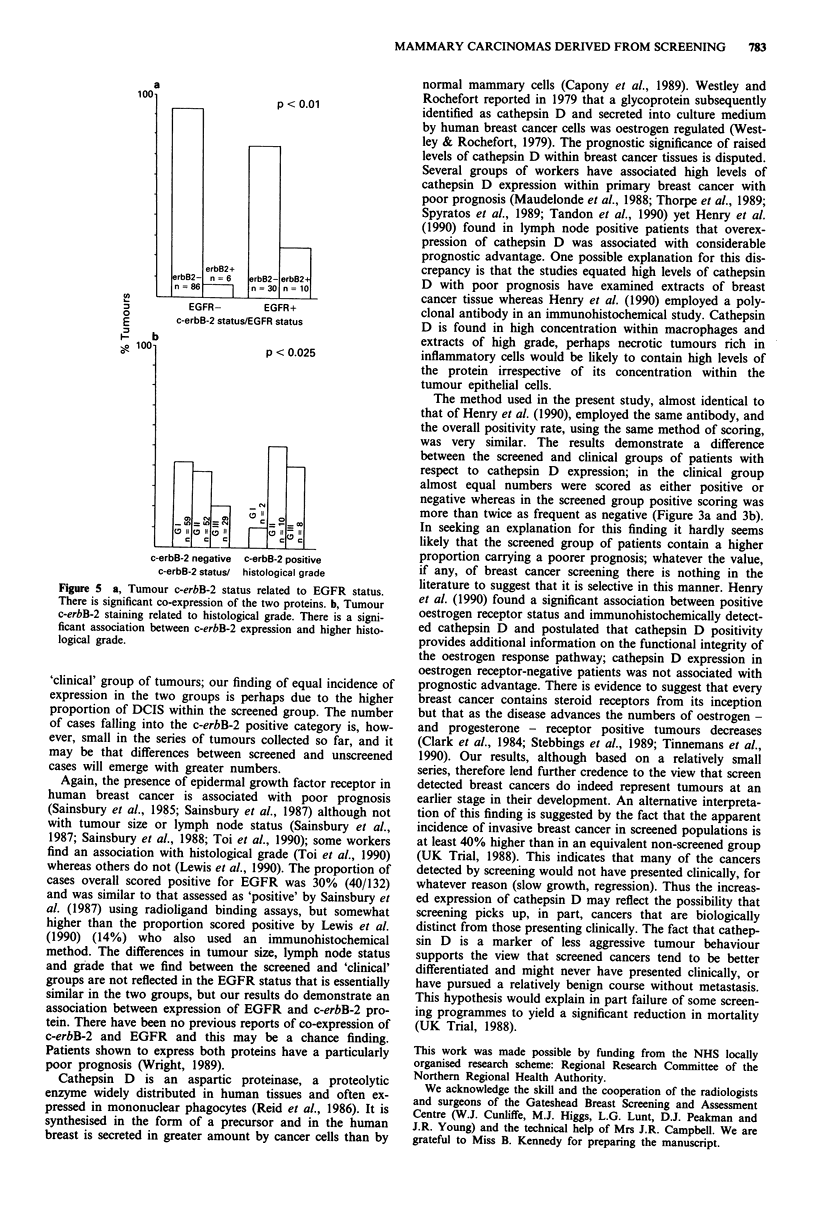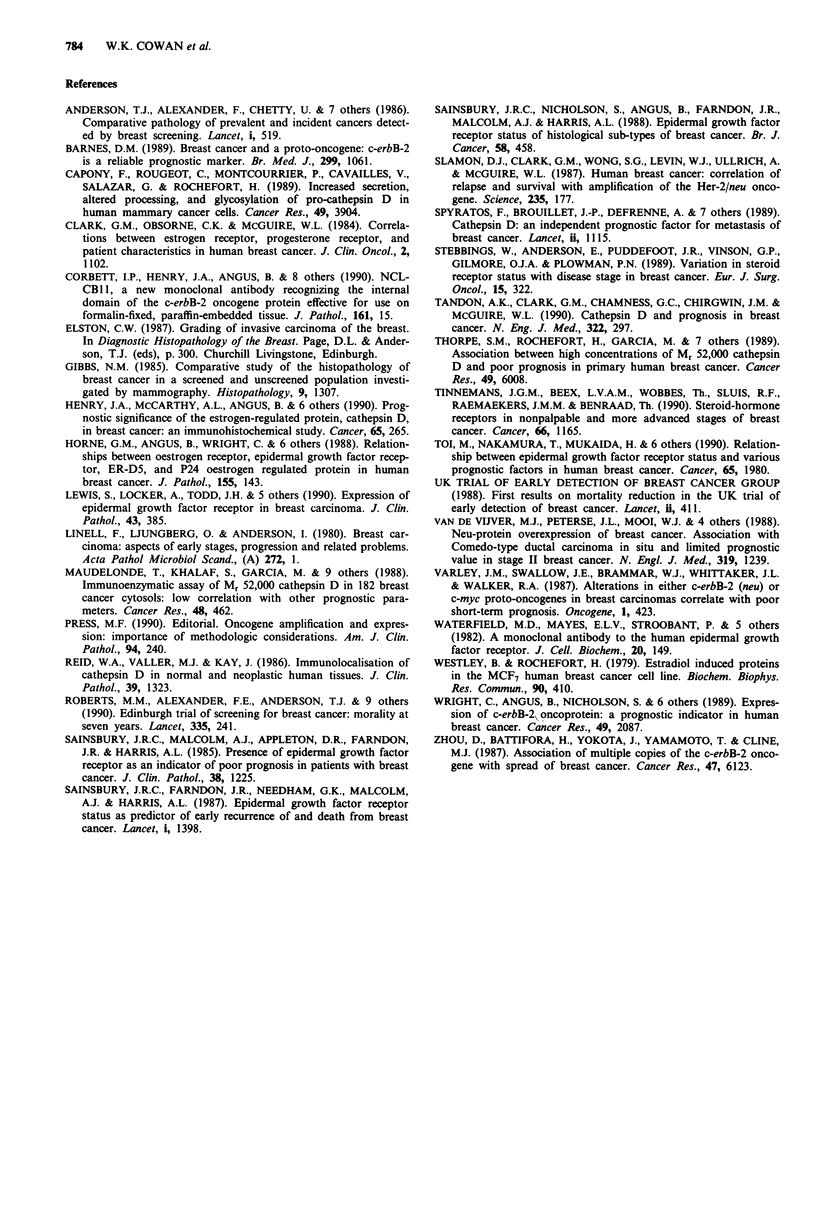# Immunohistochemical and other features of breast carcinomas presenting clinically compared with those detected by cancer screening.

**DOI:** 10.1038/bjc.1991.398

**Published:** 1991-10

**Authors:** W. K. Cowan, B. Angus, J. Henry, I. P. Corbett, W. A. Reid, C. H. Horne

**Affiliations:** Department of Pathology, Queen Elizabeth Hospital, Gateshead, UK.

## Abstract

**Images:**


Br. J. Cancer (1991), 64, 780-784 © Macmillan Press Ltd., 1991

### Immunohistochemical and other features of breast carcinomas presenting clinically compared with those detected by cancer screening

W.K. Cowan[1], B. Angus[2], J. Henry[2], I.P. Corbett[2], W.A. Reid[3] & C.H.W. Horne[2]

[1]*Department of Pathology, Queen Elizabeth Hospital, Gateshead NE9 6SX;* [2]*Department of Pathology, Royal Victoria Infirmary, Newcastle upon Tyne NE1 4LP;* [3]*Department of Pathology, University of Leeds, UK.*

**Summary** Features of 111 mammary carcinomas derived from breast cancer screening were compared with those of 69 carcinomas presenting 'clinically'. Screen detected cancers were smaller, had less likelihood of nodal metastases, included a higher proportion of *in situ* tumours and if invasive, tended to be of lower grade. Using immunohistochemical methods, the expression of c-*erb*B-2 oncoprotein, epidermal growth factor receptor (EGFR) and cathepsin D were compared in the two groups. A similar proportion of screened and unscreened tumours expressed c-*erb*B-2 oncoprotein and EGFR but expression of the oestrogen regulated protein cathepsin D was significantly more frequent in the screened group ($P < 0.05$). Although a relatively small series, the results suggest a biological difference between 'screened' and 'clinical' tumours.

Whether or not breast cancer screening results in a reduction in mortality from the disease, it is clear from several studies that screening yields tumours that are smaller, more likely to be *in situ* and clinically less advanced (Linell *et al.*, 1980; Gibbs, 1985; Anderson *et al.*, 1986; Roberts *et al.*, 1990). The question arises as to whether screen detected cancers differ biologically from those presenting clinically; the aim of this study was to determine whether the two groups differed in their expression of antigens that are associated with differing biological behaviour in terms of prognosis. Three antigens, currently of interest as prognostic indicators were chosen. Amplification of c-*erb*B-2 is regarded as a reliable prognostic marker in breast cancer (Barnes, 1989), epidermial growth factor receptor (EGFR) correlates strongly with a short disease free interval (Lewis *et al.*, 1990) and positive immunostaining for the oestrogen regulated protein cathepsin D is associated with prognostic advantage (Henry *et al.*, 1990).

### Materials and methods

Tumour tissue from 180 carcinomas was studied. Of these, 69 tumours derived from clinical presentation whereas 111 tumours were detected by breast cancer screening. The latter group comprised 80 tumours detected by population screening (UK National Health Service Breast-Screening Programme) and 31 tumours via a charity-sponsored breast screening service. The symptomatic tumours were from consecutive patients investigated and treated at the same hospital over the same period of time as those of the screened group.

Specimens were obtained immediately after removal, measured and described, and when possible a portion of tumour snap frozen and stored at −70°C. Representative blocks were fixed immediately in 10% buffered formol saline and one block post fixed in mercuric chloride.

All invasive tumours (ductal and lobular) were graded by Elston's modification of the Bloom and Richardson method (Elston, 1987). The immunohistochemical preparations were independently scored by two observers without knowledge of the derivation of the tumours; discrepancies were resolved by conference with a two headed microscope.

### *Immunohistochemistry*

*c-*erb*B-2 oncoprotein* Paraffin sections of tissue postfixed in mercuric chloride were cut at 4 μ, dewaxed and rehydrated. After treatment with 0.5% hydrogen peroxide in methanol for 10 min the sections were washed and placed in TRIS-buffered saline (TBS) for 5 min. They were then covered with 1/5 normal swine serum for 10 min after which excess serum was removed and replaced by mouse monoclonal antibody (NCL-CB11 [Corbett *et al.*, 1990]) diluted 1:20. The sections were incubated in a humid chamber at 4°C overnight. After two changes of TBS they were incubated for 30 min with biotinylated sheep-anti-mouse serum (Amersham) and streptavidin-biotin peroxidase complex for 30 min, being washed with TBS between stages. Diaminobenzidene was used as a chromagen with copper sulphate as enhancement and the sections were counterstained with Carazzi's haematoxylin.

Tumours were scored by assessment of intensity of membrane associated staining and the proportion of tumour cells stained, as previously described (Wright *et al.*, 1989), and thus placed in three categories: 2, strong staining in at least 50% of tumours cells; 1, any positive staining less than 2; 0, no staining.

*EGFR* Frozen sections (5 μ) were cut at 4°C and dried overnight at room temperature (RT). After fixation in acetone for 10 min at RT the tissue was incubated with 1/5 normal swine serum for 10 min before incubation for 30 min, with monoclonal anti-EGFR (EGF-R1 [Waterfield *et al.*, 1982], Amersham) diluted 1/40 in 1/5 normal swine serum. The sections were then incubated for 30 min with biotinylated sheep-anti-mouse serum (Amersham) and streptavidin-biotin peroxidase complex for 30 min, being washed with TBS between stages. The chromagen and counterstain were as for the c-*erb*B-2 method above. A section of human placenta was used as a control.

Tumours were scored on a basis of intensity and distribution of staining as previously described (Horne, 1987). Both membrane and cytoplasmic staining was considered. Carcinomas showing definite labelling of greater than 25% of tumour cells were regarded as 'positive'.

*Cathepsin D* Paraffin sections were cut at 4 μ, dewaxed, rehydrated and treated with hydrogen peroxide in methanol as for c-*erb*B-2 above. They were next treated with 0.1% trypsin at 37°C for 10 min and washed in water. After rinsing in TBS and incubation with normal 1/5 swine serum for 10 min as above, they were incubated with the primary antiserum (anti-cathepsin D [Reid *et al.*, 1986]) diluted 1:400 for 30 min at RT. After two changes of TBS they were incubated with biotinylated donkey-anti-rabbit serum (Amersham) and streptavidin-biotin peroxidase complex for 30 min, being washed with TBS between stages. The chromagen and counterstain were as for the c-*erb*B-2 method above.

Tumours were scored for intensity of staining as previously described (Henry *et al.*, 1990) and placed in three grades, 0, no cell staining; 1, moderate staining; 2, strong staining.

Correspondence: W.K. Cowan, Department of Pathology, Queen Elizabeth Hospital, Sheriff Hill, Gateshead, Tyne & Wear NE9 6SX, UK.
Received 4 January 1991; and in revised form 29 May 1991.

Staining of macrophages was ignored. For this study tumour staining of grade 1 or 2 was regarded as positive for purposes of analysis.

### Results

*Size, grade, invasion status, lymph node status*

Our results demonstrate certain differences between screen detected cancers and those presenting clinically. Tumours within the screened group were more often of small size; 37% of screened tumours measured less than 15 mm compared with 15% of clinical tumours ($P < 0.005$, Figure 2c). Further, whereas tumour grades were fairly evenly distributed in the clinical group, the screened group contained relatively few grade III lesions ($P < 0.025$, Figure 2d). Differences were also evident in the invasion status, *in situ* disease being uncommon (3%) in the clinical cases yet comprising 16% of screened tumours ($P < 0.05$, Figure 2b). This difference is reflected in the lymph node status where 35% of the clinical cases had axillary metastases compared with 14% of screened patients (Figure 2a).

*Immunohistochemistry*

Immunostaining for EGFR using monoclonal antibody EGFR1 showed generally a cytoplasmic pattern, although in some cases membrane associated labelling was observed (Figure 1a). Immunostaining for c-*erb*B-2 using monoclonal antibody NCL-CB11 gave, in all positive cases, intense membrane associated staining (Figure 1b). Using the polyclonal antiserum, for cathepsin D a granular cytoplasmic staining pattern was observed (Figure 1c).

Comparing screened and unscreened tumours, there was a significant difference in the expression of cathepsin D between the two groups. Almost equal positive and negative scores were recorded in the clinical group, whereas there was a marked tendency towards cathepsin D positivity in the screened group ($P < 0.05$, Figure 3a). Eliminating non-invasive carcinomas from the analysis and considering invasive carcinomas only, an excess of cathepsin D positive tumours was observed in the screened group, although falling just short of significance (Figure 3b). A similar proportion of patients in the screened and unscreened groups expressed EGFR and c-*erb*B-2 (Figure 4a and b).

Invasion status did not relate to cathepsin D expression or EGFR expression. Whilst a higher proportion of *in situ* tumours were c-*erb*B-2 positive (5/19:‘ 26%) compared to invasive lesions (20/139: 14%), this did not achieve significance. There was a significant tendency towards co-expression of c-*erb*B-2 and EGFR ($P < 0.01$, Figure 5a). There was a significant association between high histological grade and positive c-*erb*B-2 status (Figure 5b).

### Discussion

The patients within our screen detected group were largely from a prevalence screen, i.e. the first screen a patient has, detecting all prevalent disease. As expected, this yielded a number of large tumours, often palpable, measuring up to 60 mm and other examples of advanced disease. All but four the tumours were clinically stage I or II. None had evident distant metastases but skin was locally involved in two patients from each group. Despite this bias, diluting the screened group with tumours that would not be expected within second and subsequent (incidence) screens, our results demonstrate certain differences between the screen detected cancers and those presenting clinically. The differences relating to smaller size, lower grade, higher incidence of *in situ* disease and lower incidence of lymph node metastases within the screened group are in accordance with previous work (Gibbs, 1985; Anderson *et al.*, 1986).

Overexpression of c-*erb*B-2 oncoprotein is regarded by

**Figure 1** **a**, Invasive ductal carcinoma of breast: immunohistochemical staining for EGFR using monoclonal antibody EGFR1. Note both cytoplasmic and membrane associated labelling. **b**, Invasive ductal carcinoma of breast: immunohistochemical staining for c-*erb*B-2 oncoprotein using monoclonal antibody NCL-CB11. Note intense membrane associated staining of tumour cells and absence of staining of non-neoplastic epithelium (centre, left). **c**, Invasive ductal carcinoma of breast: immunohistochemical staining for cathepsin D using rabbit polyclonal antiserum. Note the fine granular cytoplasmic staining for this lysosomal enzyme.

**Figure 2** Presentation mode related to lymph node status **a**, invasion status **b**, tumour size **c** and tumour histological grade **d**. Screened cancers tend to be lymph node negative, non-invasive, small and of low histological grade, compared to the non-screened group.

**Figure 3** Presentation mode related to cathepsin D expression. Note the excess of cathepsin D positive tumours in the screened group: **a**, all cases, **b**, invasive carcinomas only.

**Figure 4** Presentation mode related to c-*erb*B-2 expression. The proportion of c-*erb*B-2 positive cases in each group is similar: **a**, all cases, **b**, invasive carcinomas only.

most workers to equate with poor prognosis (Barnes, 1989; Slamon *et al.*, 1987; Wright *et al.*, 1989; Varley *et al.*, 1987; Zhou *et al.*, 1987) although this is not a universal finding, perhaps due to differences in methodology between authors (Press, 1990). In our experience, staining for c-*erb*B-2 protein is enhanced by post fixation of the formalin-fixed tissue by mercuric chloride; without standardisation of methods, rates of expression cannot be compared between different groups of workers. There is general agreement, however, that over-expression is frequently found in DCIS of the comedo, large cell type (van de Vijver *et al.*, 1988). It might have been expected that overexpression of c-*erb*B-2 protein, associated as it is with poorer prognosis, would be more frequent in our

**Figure 5** **a**, Tumour c-*erb*B-2 status related to EGFR status. There is significant co-expression of the two proteins. **b**, Tumour c-*erb*B-2 staining related to histological grade. There is a significant association between c-*erb*B-2 expression and higher histological grade.

'clinical' group of tumours; our finding of equal incidence of expression in the two groups is perhaps due to the higher proportion of DCIS within the screened group. The number of cases falling into the c-*erb*B-2 positive category is, however, small in the series of tumours collected so far, and it may be that differences between screened and unscreened cases will emerge with greater numbers.

Again, the presence of epidermal growth factor receptor in human breast cancer is associated with poor prognosis (Sainsbury *et al.*, 1985; Sainsbury *et al.*, 1987) although not with tumour size or lymph node status (Sainsbury *et al.*, 1987; Sainsbury *et al.*, 1988; Toi *et al.*, 1990); some workers find an association with histological grade (Toi *et al.*, 1990) whereas others do not (Lewis *et al.*, 1990). The proportion of cases overall scored positive for EGFR was 30% (40/132) and was similar to that assessed as 'positive' by Sainsbury *et al.* (1987) using radioligand binding assays, but somewhat higher than the proportion scored positive by Lewis *et al.* (1990) (14%) who also used an immunohistochemical method. The differences in tumour size, lymph node status and grade that we find between the screened and 'clinical' groups are not reflected in the EGFR status that is essentially similar in the two groups, but our results do demonstrate an association between expression of EGFR and c-*erb*B-2 protein. There have been no previous reports of co-expression of c-*erb*B-2 and EGFR and this may be a chance finding. Patients shown to express both proteins have a particularly poor prognosis (Wright, 1989).

Cathepsin D is an aspartic proteinase, a proteolytic enzyme widely distributed in human tissues and often expressed in mononuclear phagocytes (Reid *et al.*, 1986). It is synthesised in the form of a precursor and in the human breast is secreted in greater amount by cancer cells than by normal mammary cells (Capony *et al.*, 1989). Westley and Rochefort reported in 1979 that a glycoprotein subsequently identified as cathepsin D and secreted into culture medium by human breast cancer cells was oestrogen regulated (Westley & Rochefort, 1979). The prognostic significance of raised levels of cathepsin D within breast cancer tissues is disputed. Several groups of workers have associated high levels of cathepsin D expression within primary breast cancer with poor prognosis (Maudelonde *et al.*, 1988; Thorpe *et al.*, 1989; Spyratos *et al.*, 1989; Tandon *et al.*, 1990) yet Henry *et al.* (1990) found in lymph node positive patients that overexpression of cathepsin D was associated with considerable prognostic advantage. One possible explanation for this discrepancy is that the studies equated high levels of cathepsin D with poor prognosis have examined extracts of breast cancer tissue whereas Henry *et al.* (1990) employed a polyclonal antibody in an immunohistochemical study. Cathepsin D is found in high concentration within macrophages and extracts of high grade, perhaps necrotic tumours rich in inflammatory cells would be likely to contain high levels of the protein irrespective of its concentration within the tumour epithelial cells.

The method used in the present study, almost identical to that of Henry *et al.* (1990), employed the same antibody, and the overall positivity rate, using the same method of scoring, was very similar. The results demonstrate a difference between the screened and clinical groups of patients with respect to cathepsin D expression; in the clinical group almost equal numbers were scored as either positive or negative whereas in the screened group positive scoring was more than twice as frequent as negative (Figure 3a and 3b). In seeking an explanation for this finding it hardly seems likely that the screened group of patients contain a higher proportion carrying a poorer prognosis; whatever the value, if any, of breast cancer screening there is nothing in the literature to suggest that it is selective in this manner. Henry *et al.* (1990) found a significant association between positive oestrogen receptor status and immunohistochemically detected cathepsin D and postulated that cathepsin D positivity provides additional information on the functional integrity of the oestrogen response pathway; cathepsin D expression in oestrogen receptor-negative patients was not associated with prognostic advantage. There is evidence to suggest that every breast cancer contains steroid receptors from its inception but that as the disease advances the numbers of oestrogen – and progesterone – receptor positive tumours decreases (Clark *et al.*, 1984; Stebbings *et al.*, 1989; Tinnemans *et al.*, 1990). Our results, although based on a relatively small series, therefore lend further credence to the view that screen detected breast cancers do indeed represent tumours at an earlier stage in their development. An alternative interpretation of this finding is suggested by the fact that the apparent incidence of invasive breast cancer in screened populations is at least 40% higher than in an equivalent non-screened group (UK Trial, 1988). This indicates that many of the cancers detected by screening would not have presented clinically, for whatever reason (slow growth, regression). Thus the increased expression of cathepsin D may reflect the possibility that screening picks up, in part, cancers that are biologically distinct from those presenting clinically. The fact that cathepsin D is a marker of less aggressive tumour behaviour supports the view that screened cancers tend to be better differentiated and might never have presented clinically, or have pursued a relatively benign course without metastasis. This hypothesis would explain in part failure of some screening programmes to yield a significant reduction in mortality (UK Trial, 1988).

This work was made possible by funding from the NHS locally organised research scheme: Regional Research Committee of the Northern Regional Health Authority.

We acknowledge the skill and the cooperation of the radiologists and surgeons of the Gateshead Breast Screening and Assessment Centre (W.J. Cunliffe, M.J. Higgs, L.G. Lunt, D.J. Peakman and J.R. Young) and the technical help of Mrs J.R. Campbell. We are grateful to Miss B. Kennedy for preparing the manuscript.

### References

ANDERSON, T.J., ALEXANDER, F., CHETTY, U. & 7 others (1986). Comparative pathology of prevalent and incident cancers detected by breast screening. *Lancet*, **i**, 519.

BARNES, D.M. (1989). Breast cancer and a proto-oncogene: c-*erb*B-2 is a reliable prognostic marker. *Br. Med. J.*, **299**, 1061.

CAPONY, F., ROUGEOT, C., MONTCOURRIER, P., CAVAILLES, V., SALAZAR, G. & ROCHEFORT, H. (1989). Increased secretion, altered processing, and glycosylation of pro-cathepsin D in human mammary cancer cells. *Cancer Res.*, **49**, 3904.

CLARK, G.M., OBSORNE, C.K. & MCGUIRE, W.L. (1984). Correlations between estrogen receptor, progesterone receptor, and patient characteristics in human breast cancer. *J. Clin. Oncol.*, **2**, 1102.

CORBETT, I.P., HENRY, J.A., ANGUS, B. & 8 others (1990). NCL-CB11, a new monoclonal antibody recognizing the internal domain of the c-*erb*B-2 oncogene protein effective for use on formalin-fixed, paraffin-embedded tissue. *J. Pathol.*, **161**, 15.

ELSTON, C.W. (1987). Grading of invasive carcinoma of the breast. In *Diagnostic Histopathology of the Breast*. Page, D.L. & Anderson, T.J. (eds), p. 300. Churchill Livingstone, Edinburgh.

GIBBS, N.M. (1985). Comparative study of the histopathology of breast cancer in a screened and unscreened population investigated by mammography. *Histopathology*, **9**, 1307.

HENRY, J.A., MCCARTHY, A.L., ANGUS, B. & 6 others (1990). Prognostic significance of the estrogen-regulated protein, cathepsin D, in breast cancer: an immunohistochemical study. *Cancer*, **65**, 265.

HORNE, G.M., ANGUS, B., WRIGHT, C. & 6 others (1988). Relationships between oestrogen receptor, epidermal growth factor receptor, ER-D5, and P24 oestrogen regulated protein in human breast cancer. *J. Pathol.*, **155**, 143.

LEWIS, S., LOCKER, A., TODD, J.H. & 5 others (1990). Expression of epidermal growth factor receptor in breast carcinoma. *J. Clin. Pathol.*, **43**, 385.

LINELL, F., LJUNGBERG, O. & ANDERSON, I. (1980). Breast carcinoma: aspects of early stages, progression and related problems. *Acta Pathol Microbiol Scand.*, (A) **272**, 1.

MAUDELONDE, T., KHALAF, S., GARCIA, M. & 9 others (1988). Immunoenzymatic assay of $M_r$ 52,000 cathepsin D in 182 breast cancer cytosols: low correlation with other prognostic parameters. *Cancer Res.*, **48**, 462.

PRESS, M.F. (1990). Editorial. Oncogene amplification and expression: importance of methodologic considerations. *Am. J. Clin. Pathol.*, **94**, 240.

REID, W.A., VALLER, M.J. & KAY, J. (1986). Immunolocalisation of cathepsin D in normal and neoplastic human tissues. *J. Clin. Pathol.*, **39**, 1323.

ROBERTS, M.M., ALEXANDER, F.E., ANDERSON, T.J. & 9 others (1990). Edinburgh trial of screening for breast cancer: morality at seven years. *Lancet*, **335**, 241.

SAINSBURY, J.R.C., MALCOLM, A.J., APPLETON, D.R., FARNDON, J.R. & HARRIS, A.L. (1985). Presence of epidermal growth factor receptor as an indicator of poor prognosis in patients with breast cancer. *J. Clin. Pathol.*, **38**, 1225.

SAINSBURY, J.R.C., FARNDON, J.R., NEEDHAM, G.K., MALCOLM, A.J. & HARRIS, A.L. (1987). Epidermal growth factor receptor status as predictor of early recurrence of and death from breast cancer. *Lancet*, **i**, 1398.

SAINSBURY, J.R.C., NICHOLSON, S., ANGUS, B., FARNDON, J.R., MALCOLM, A.J. & HARRIS, A.L. (1988). Epidermal growth factor receptor status of histological sub-types of breast cancer. *Br. J. Cancer*, **58**, 458.

SLAMON, D.J., CLARK, G.M., WONG, S.G., LEVIN, W.J., ULLRICH, A. & MCGUIRE, W.L. (1987). Human breast cancer: correlation of relapse and survival with amplification of the Her-2/*neu* oncogene. *Science*, **235**, 177.

SPYRATOS, F., BROUILLET, J.-P., DEFRENNE, A. & 7 others (1989). Cathepsin D: an independent prognostic factor for metastasis of breast cancer. *Lancet*, **ii**, 1115.

STEBBINGS, W., ANDERSON, E., PUDDEFOOT, J.R., VINSON, G.P., GILMORE, O.J.A. & PLOWMAN, P.N. (1989). Variation in steroid receptor status with disease stage in breast cancer. *Eur. J. Surg. Oncol.*, **15**, 322.

TANDON, A.K., CLARK, G.M., CHAMNESS, G.C., CHIRGWIN, J.M. & MCGUIRE, W.L. (1990). Cathepsin D and prognosis in breast cancer. *N. Eng. J. Med.*, **322**, 297.

THORPE, S.M., ROCHEFORT, H., GARCIA, M. & 7 others (1989). Association between high concentrations of $M_r$ 52,000 cathepsin D and poor prognosis in primary human breast cancer. *Cancer Res.*, **49**, 6008.

TINNEMANS, J.G.M., BEEX, L.V.A.M., WOBBES, Th., SLUIS, R.F., RAEMAEKERS, J.M.M. & BENRAAD, Th. (1990). Steroid-hormone receptors in nonpalpable and more advanced stages of breast cancer. *Cancer*, **66**, 1165.

TOI, M., NAKAMURA, T., MUKAIDA, H. & 6 others (1990). Relationship between epidermal growth factor receptor status and various prognostic factors in human breast cancer. *Cancer*, **65**, 1980.

UK TRIAL OF EARLY DETECTION OF BREAST CANCER GROUP (1988). First results on mortality reduction in the UK trial of early detection of breast cancer. *Lancet*, **ii**, 411.

VAN DE VIJVER, M.J., PETERSE, J.L., MOOI, W.J. & 4 others (1988). Neu-protein overexpression of breast cancer. Association with Comedo-type ductal carcinoma in situ and limited prognostic value in stage II breast cancer. *N. Engl. J. Med.*, **319**, 1239.

VARLEY, J.M., SWALLOW, J.E., BRAMMAR, W.J., WHITTAKER, J.L. & WALKER, R.A. (1987). Alterations in either c-*erb*B-2 (*neu*) or c-*myc* proto-oncogenes in breast carcinomas correlate with poor short-term prognosis. *Oncogene*, **1**, 423.

WATERFIELD, M.D., MAYES, E.L.V., STROOBANT, P. & 5 others (1982). A monoclonal antibody to the human epidermal growth factor receptor. *J. Cell. Biochem.*, **20**, 149.

WESTLEY, B. & ROCHEFORT, H. (1979). Estradiol induced proteins in the $MCF_7$ human breast cancer cell line. *Biochem. Biophys. Res. Commun.*, **90**, 410.

WRIGHT, C., ANGUS, B., NICHOLSON, S. & 6 others (1989). Expression of c-*erb*B-2 oncoprotein: a prognostic indicator in human breast cancer. *Cancer Res.*, **49**, 2087.

ZHOU, D., BATTIFORA, H., YOKOTA, J., YAMAMOTO, T. & CLINE, M.J. (1987). Association of multiple copies of the c-*erb*B-2 oncogene with spread of breast cancer. *Cancer Res.*, **47**, 6123.